# Modelling the performance of USV manoeuvring and target tracking: an approach using frequency modulated continuous wave radar rotary system

**DOI:** 10.1186/2193-1801-2-184

**Published:** 2013-04-24

**Authors:** Chiemela Onunka, Remigius Chidozie Nnadozie

**Affiliations:** Faculty of Engineering, Mangosuthu University of Technology, Umlazi, Durban, South Africa

## Abstract

The performance of frequency modulated continuous wave (FMCW) radar in tracking targets is presented and analysed. Obstacle detection, target tracking and radar target tracking performance models are developed and were used to investigate and to propose ways of improving the autonomous motion of unmanned surface vehicle (USV). Possible factors affecting the performance of FMCW radar in tracking targets are discussed and analysed.

**Electronic supplementary material:**

The online version of this article (doi:10.1186/2193-1801-2-184) contains supplementary material, which is available to authorized users.

## Introduction

There have been growing interests in the use of frequency modulated continuous wave radar systems in USV applications such as autonomous navigation, target ranging and direction (Chan & Judah 1998). Target range determination can be performed by means of well adapted frequency modulated continuous wave (FMCW) system. With FMCW, target range measurements can be made over large frequency bandwidth and FMCW system provides less accurate and unambiguous range measurements (Reinhard & Schiek 1997). In recent advancements, ultra-wideband frequency modulated continuous wave (UWBFMCW) radar systems have been developed to take advantage of microwave technology in providing simple solutions that can be adapted to suite the duration of modulation especially in tracking moving targets (Maaref et al. 2009). In recent developments, it has become a common practice to monitor coastlines with high frequency surface wave (HFSW) radar for sensing and monitoring ocean surface characteristics. The radar algorithms developed over the years were based on studies investigating the interaction between the ocean surface and radar signals. To achieve wider operating radar bandwidths for ocean scanning and target tracking, linear frequency modulation waveforms (LFMW) are employed in such radar systems (Mahafza & Elsherbeni 2004). Due to the high positioning accuracy and robustness of FMCW radar (Subramanian et al. 2011), remote sensing of coastal lines using frequency surface wave radar was studied using FMCW as its primary wave form. This facilitated the extraction of target information on or close to the surface of the ocean using FMCW (Zhang et al. 2008). Obtaining target range autofocus characteristics required the critical consideration of the radar signal-to-noise ratio (SNR), radar dynamic range properties and geometric precision (Scheiblhofer et al. 2006). The linearity of radar autofocus characteristics can be achievable using phase-gradient algorithm and time-domain warping of de-chirped radar signal (Middleton et al. 2011). The non-coherent nature of radar systems has facilitated the development and integration low cost of radar sensors without the synchronisation of radar frequency or radar signal phase. Radar applications in autonomous navigation of unmanned surface vehicles (USVs) have introduced the classification of targets which are based on target range, cross-range, and target velocity and radar power. This paper models and investigates the possible factors that affects the performance of FMCW radar in USV manoeuvring and target tracking. The description of radar waveform is provided. This is followed by obstacle manoeuvring and target tracking which provides insight to performance monitoring of the radar in detecting and tracking obstacles. Finally the performance of the radar is evaluated in the presence of targets and noise while considering the effects of radar sweep, power and the frequency of the waveform.

## FMCW radar wave form

The choice of wave form and signal processing methodology in USV radar systems implementation was dependent on the type of mission assigned to the radar and the specific USV mission and function. The complexity and price associated to the development of USV systems constituted the major factor in deciding the type of radar waveform, hardware and software that were suitable for the specific functions that were assigned to the USV. Radar systems have the advantage of using continuous wave forms with or without frequency modulation. The frequency modulation of radar systems can be implemented either through analog techniques or digital methods. An important consideration in the modulation of radar frequencies was the radar range and Doppler frequencies as these had direct associations to the choice of waveform frequency properties implemented in the radar system (Mahafza & Elsherbeni 2010). FMCW radar band pass signal *x*(*t*) can be presented as:

1 $$x\left(t\right)=r\left(t\right)\cos\left(2\pi f_{o}t+\Phi_{x}\left(t\right)\right)$$

Where *r*(*t*) represents the radar signal amplitude modulation or envelop, Φ_*x*_(*t*) represents the radar signal phase modulation and *f*_*o*_  represents the radar carrier frequency. The radar frequency modulation was modelled as:

2 $$f_{m}\left(t\right)=\frac{1}{2\pi }\;\frac{d}{\mathrm{dt}}\;\Phi_{x}\left(t\right)$$

The radar signal instantaneous frequency is modelled as:

3 $$f_{i}\left(t\right)=f_{0}+f_{m}\left(t\right)\;$$

The radar signal *x*(*t*) may also be represented as an analytic signal forming the real part of the complex signal *ψ*(*t*) illustrated in equation (4)

4 xt=Reψt=RertejΦxtej2πfot

The radar analytic signal is then defined as:

5 $$\psi \left(t\right)=v\left(t\right)e^{j2\pi f_{o}t}$$

Where

6 $$v\left(t\right)=r\left(t\right)e^{j\Phi_{x}\left(t\right)}$$

Implementing a Fourier transform to equation (5) while keeping the signal under the following conditions illustrated in equation (7) yields:

7 $$\Psi \left(\omega \right)=\left[\begin{array}{c} 2X\left(\omega \right)\\ 0 \end{array}\right]\;\begin{array}{c} \omega \;\geq \;0\\ \omega \;<\;0 \end{array}$$

Where Ψ(*ω*) represents the Fourier transform of *ψ*(*t*), *ω* = 2*πf*_0_ and *X*(*ω*) = *ψ*(*t*) is the Fourier transform of  *x*(*t*). Implementing a step function to equation (7) yields:

8 $$\Psi \left(\omega \right)=2U\left(\omega \right)X\left(\omega \right)$$

Where *U*(*ω*) represents the step function of the radar signal in frequency domain. From the above models, it can be shown that *ψ*(*t*)  is represented as:

9 $$\psi \left(t\right)=x\left(t\right)+j\tilde{x}\left(t\right)$$

Where $\tilde{x}\left(t\right)$ represents the Hilbert transform of the radar signal  *x*(*t*). The energy associated with radar signal *x*(*t*) can be illustrated using Parseval’s theorem (Mahafza & Elsherbeni 2010) as indicated below,

10 $$E_{x}=\frac{1}{2}\;\overset{\infty }{\underset{-\infty }{\int }}x^{2}\left(t\right)\mathrm{dt}=\frac{1}{2}\;E_{\psi }$$

The exponential form of the radar signal can be modelled as (Zhang et al. 2008):

11 $$i\left(t_{r}\right)=I_{o}e^{j\left(\omega_{o}t_{r}+\alpha \pi t_{r}^{2}\right)}\;-\;\frac{T_{r}}{2}\leq t_{r}<\;\frac{T_{r}}{2}$$

Where  *I*_*o*_ represents the current supply to the radar, *ω*_*o*_ represents the central radian frequency of the radar waveform and  *ω*_*o*_ = 2*πf*_0_, *α* represents the radar sweep frequency rate in Hz/s, *t*_*r*_ represents the time variable and *T*_*r*_  represents the radar sweep frequency interval. The radar frequency modulation sweep rate is the ratio of the radar frequency bandwidth *B* and sweep interval. Hence radar frequency rate is modelled as:

12 $$\alpha =\frac{B}{T_{r}}$$

The continuous variation in the transmitted frequency of FMCW radar at any given time with the difference in transmitted and received radar signal defines the radar beat frequency  *f*_*b*_. The radar beat frequency portrays the characteristic property and measure of target range *R* and it is modelled as (Chan & Judah 1998):

13 $$R=\frac{cf_{b}}{2f_{m}}$$

Where *c* represents the speed of light in air, *f*_*b*_ represents the radar beat frequency and *f*_*m*_ = ∆*f/T* represents the rate of change of the transmitted frequency. The sweep frequency of the radar source is denoted by ∆*f* and *T* denotes the time taken for each radar signal source sweep (Skolink 1981). The radar echo mixed with some portion of the transmitted signal received after *τ* seconds produces the beat frequency of the radar. The radar echo is given as (Dorp & Groen 2010):

14 $$\tau =\frac{2r_{s}}{c}$$

Where *r*_*s*_ represents the target slant range and *c* is the speed of light. The wave form transmitted by FMCW radar in compact form is given as:

15 $$s\left(t\right)=S_{o}e^{j\phi_{t}\left(t\right)}$$

Where *ϕ*_*t*_ represents the transmitted frequency phase with pulse width *T*, out power *S*_*0*_ and bandwidth *B* having an instantaneous angular frequency of

16 $$\omega_{t}\left(t\right)=\frac{\delta \phi_{t}\left(t\right)}{\mathrm{\delta t}}\;$$

The nonlinear transmitted frequency with instantaneous up-chirp phase is modelled as:

17 $$\phi_{t}\left(t\right)=2\pi \left(f_{o}t+\frac{\mu }{2}t^{2}\right)$$

With the corresponding instantaneous frequency given as:

18 $$f\left(t\right)=\frac{1}{2\pi }\;\frac{d}{\mathrm{dt}}\;\phi \left(t\right)=f_{o}+\mathrm{\mu t}$$

Similarly the radar instantaneous down-chirp phase and frequency is given as:

19 $$\phi_{t}\left(t\right)=2\pi \left(f_{o}t-\;\frac{\mu }{2}t^{2}\right)$$

20 $$f\left(t\right)=\frac{1}{2\pi }\;\frac{d}{\mathrm{dt}}\;\phi \left(t\right)=f_{o}-\mathrm{\mu t}$$

For $\;-\frac{Τ}{2}\leq t\leq \frac{Τ}{2}$, where *f*_*0*_ represents the radar centre frequency and *μ = (2πB)/T* represents frequency modulation coefficient. The radar return echo with delay *τ* modelled as *s*(*t-τ*) is mixed with the transmitted radar signal to generate the radar beat waveform *S*_*b*_.

21 $$s_{b}\left(t\right)=s\left(t\right)s^{*}\left(t-\tau \right)$$

Thus the instantaneous transmitted radar signal phase presented as (Dorp & Groen 2010):

22 $$\phi_{t}\left(t\right)=a_{0}+a_{1}t+a_{2}\;{\left(t-\frac{T_{0}}{2}\right)}^{2}$$

for 0 ≤ *t* ≤ *T*_0_

Having the beat signal phase (Dorp & Groen 2010):

23 $$\phi_{b}\left(t\right)=\phi_{t}\left(t\right)-\phi_{t}\left(t-\tau \right)$$

24 $$=b_{0}+b_{1}t+b_{2}{\left(t-\frac{T_{0}}{2}\right)}^{2}$$

for 0 ≤ *t* ≤ *T*_0_

With

25 $$b_{0}=a_{1}\tau +a_{2}\tau +2a_{2}\tau \frac{T_{0}}{2}$$

26 $$b_{1}=2a_{2}\tau$$

## Obstacle manoeuvring and target tracking model

The identification of obstacles and targets as USVs navigate on the ocean surface requires some form of target position deterministic algorithm which allows for target position and velocity estimation and determination. To perform adequate autonomous motion operation, the USV is equipped and incorporated with obstacle and target tracking filters. The radar obstacle tracker provides the required optimum estimates of target position and velocity which are incorporated in the dynamic model on which the target tracking filter is based on. The target tracking filter provides an accurate representation of the actual nature of obstacle motion path. The target tracking model is developed based on the assumption that the target manoeuvres at a constant velocity in which the straight line obstacle trajectory will be lost eventually should the obstacle manoeuvre in a different path. The target manoeuvring algorithm interacts with other USV autonomous motion deterministic algorithms in probabilistic models and it is based on Bar-Shalom’s (Bar-Shalom & Cilang 1989; Bar-Shalom & Birmiwal 1982) interacting multiple model (IMM) algorithm. In the absence of motion characteristic properties of the obstacle, the obstacle is modelled as an object moving with constant velocity in a plane with the addition of process noise to incorporate slight changes in the target velocity. The resulting dormant target model discretized over time *T* in the Cartesian coordinates is represented as:

27 $$x\left(k+1\right)=\mathrm{Fx}\left(k\right)+G\;w\left(k\right)$$

Where

28 $$x=\left[\begin{array}{c} x\\ \begin{array}{c} \dot{x}\\ \begin{array}{c} y\\ \dot{y} \end{array} \end{array} \end{array}\right]$$

29 $$F=\left[\begin{array}{c} \begin{array}{cc} 1 & T\\ 0 & 1 \end{array}\;\begin{array}{cc} 0 & 0\\ 0 & 0 \end{array}\\ \begin{array}{cc} 0 & 0\\ 0 & 0 \end{array}\;\begin{array}{cc} 0 & T\\ 0 & 1 \end{array} \end{array}\;\right]$$

30 $$w=\left[\begin{array}{c} w_{1}\\ w_{2} \end{array}\right]$$

31 $$G=\left[\begin{array}{c} \begin{array}{cc} T/2 & 0\\ 1\; & 0 \end{array}\\ \begin{array}{cc} 0 & \;T/2\\ 0 & 0 \end{array} \end{array}\right]$$

With system characteristic process noise modelled as:

32 $$E\left\{w\left(k\right)\right\}=0;\;E\left\{w\left(k\right)w^{T}\left(j\right)\right\}=Q\delta_{\mathrm{kj}}$$

With system state estimate as $\hat{X}\left(0|0\right)$, system covariance as $\hat{P}\left(0|0\right)$ and *Qδ*_*kj*_ detects whether target manoeuvre has occurred (Ramachandra 2000).

## FMCW radar performance evaluation setup

To demonstrate the technique of target tracking with FMCW radar, the signal processing procedures integrated non-linear sweep frequency; bandwidth and power correction techniques which were in Fourier transform for target range tracking. The FMCW upsweep frequency was linearized by the radar waveform generator to produce the phase modulated waveform modelled as:

33 $$s_{1}\left(t\right)=a_{1}\mathrm{cos\phi}\left(t\right)$$

And

34 $$\phi \left(t\right)=2\pi f_{0}t+\pi \frac{B}{T_{m}}t^{2}$$

Where *ϕ*(*t*) represents the radar phase at an interval *t*, *f*_*0*_ represents the carrier frequency of the radar, *B* denotes the radar sweep width and *T*_*m*_ denotes the radar frequency pulse repetition period. The instantaneous transmitted waveform frequency is given as:

35 $$F\left(t\right)=\frac{1}{2\pi }\frac{d}{\mathrm{dt}}\phi \left(t\right)=\;f_{0}+\frac{B}{T_{m}}t$$

**Figure 1:**
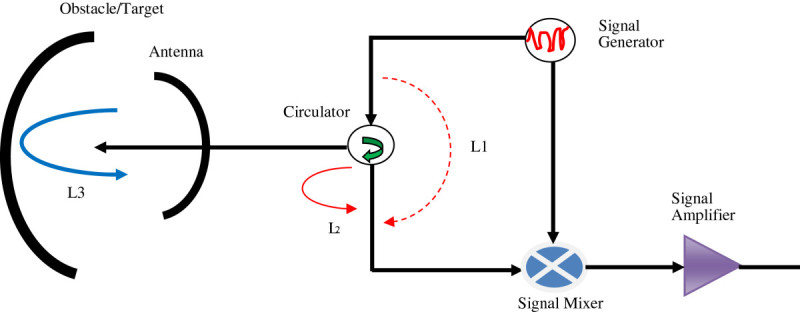
**Simplified FMCW radar circuitry.**

With the radar frequency varying linearly at an interval of (*0,T*_*m*_) between *f*_*0*_ and *f0 + B*. The FMCW radar waveform was split into two parts and passed into the radar mixer. The first part of the radar signal which is relatively small was used as a reference signal to facilitate the detection of echo signals. The larger part of the radar waveform was passed to the circulator through to the radar antenna. The circulator provided multiport system that allowed electric signals to be propagated in clockwise direction as illustrated in Figure 1. The circulator facilitated the sharing of signals from the transmission and receiving antennas. At the exit of the antenna, the radar waveform propagates out into the air towards an obstacle or target where it is reflected and returned by the receiving antenna. The received waveform then goes back into the circulator. An obstacle located at the distance *r* from the radar, generated an echo that is received by the radar waveform mixer in the form (Komarov & Smolskiy 2003)

36 $$s_{2}\left(t\right)=a_{2}\mathrm{cos\phi}\left(t-\tau \right)$$

Where *τ* = 2*r*/*c* represents the radar echo propagation delay, *c* is the speed of light and *a*_*2*_ accounts for radar waveform propagation losses, obstacle reflectivity and performance parameters of the FMCW. At the radar mixer, the echo signals and the radar reference signals are multiplied. The process generates sum and difference waveform frequencies. The sum frequencies were usually in the order of twice the radar carrier frequency and the radar electronic circuitry cannot accommodate such high frequencies. Thus only the difference radar waveform frequencies were allowed to pass out of the radar mixer. The waveform that came out the radar mixer was modeled as:

37 $$\begin{array}{ll} \;s_{3}\left(t\right) & =a_{3}\cos\left[\phi \left(t\right)-\phi \left(t-\tau \right)\right]\\ =a_{3}\cos\left(2\pi f_{\mathrm{beat}}t+2\pi f_{0}\tau -\frac{\mathrm{\pi B}}{T_{m}}\tau^{2}\right) \end{array}$$

Where

38 $$f_{\mathrm{beat}}=\frac{\mathrm{B\tau}}{T_{m}}=f_{m}\frac{r}{\mathrm{dr}}$$

And it represented the beat frequency of an obstacle echo. The radar pulse modulation frequency is denoted by $f_{m}=T_{m}^{-1}$ and *dr* = *c*/(2*B*) denotes the radar range resolution of the radar pulse (Brooker 2005).

## FMCW radar obstacle detection

For autonomous motion of USV radar navigation applications, obstacles are detected through the performance of Fourier analysis on the output waveform from the radar mixer. Implementing Fourier transform on the output signal with a rectangular window of integration over period of length *T*_*m*_ generates the following power in the radar mixer output waveform:

39 $$S_{\mathrm{echo}}\left(f,f_{\mathrm{beat}},B,T_{m}\right)=P_{r}{\left(\frac{\sin\left[\pi \left(f-f_{\mathrm{beat}}\right)T_{m}\right]}{\pi \left(f-f_{\mathrm{beat}}\right)T_{m}}\right)}^{2}$$

At the above model, *f* represents the radar analysis frequency and $P_{r}=a_{3}^{2}$ represents the power of the echo from the obstacle. In using the standard radar equation to model the echo power, the echo power is modeled as:

40 $$P_{r}=P_{t}\frac{\sigma G_{t}G_{r}\lambda^{2}}{{\left(4\pi \right)}^{3}r^{4}}$$

Where *P*_*t*_ denotes the radar transmission power, *σ* denotes the radar cross section, *λ* denotes the radar wavelength, *r* denotes the distance between the radar and the obstacle with *G*_*t*_ and *G*_*r*_ denoting the gains on the power scale of the transmitting and receiving antennas. In the Furuno radar used for the development of the USV, it has a single antenna and thus the gains (*G*_*t*_ *= G*_*r*_ *= G*) are equal. The use of non-uniform window of integration in the Fourier analysis of the output waveform from the radar mixer provided an effective measure in the signal analysis as it reduced side lobe effects in the spectral response of the radar signal. The implementation of a Hamming window of integration in the Fourier analysis provided means of detecting obstacles and targets that are within close range with large differences in echo level. The hamming window of integration for the Fourier analysis of the output signal from the mixer is modeled as:

41 $$\begin{array}{ll} \;S_{\mathrm{echo}}\left(f,f_{\mathrm{beat}},B,T_{m}\right)= & P_{r}\left(\frac{\sin\left[\pi \left(f-f_{\mathrm{beat}}\right)T_{m}\right]}{\pi \left(f-f_{\mathrm{beat}}\right)T_{m}}+\frac{0.92}{1.08}\right.\\ {\left.\frac{\left(f-f_{\mathrm{beat}}\right)T_{m}\sin[\pi \left(f-f_{\mathrm{beat}}\right)T_{m}}{\pi \left[1-{\left(f-f_{\mathrm{beat}}\right)}^{2}T_{m}^{2}\right]}\right)}^{2} \end{array}$$

In implementing a rectangular window of integration at the output signal from the mixer, stronger echo at 13 dB will obscure weaker echo returned from an obstacle. Thus the detection performance of the FMCW radar is limited to the signal phase noise that was propagated through the circulator. The power spectrum of the signal phase noise *(w/Hz)* is modelled as:

42 $$S_{\mathrm{gen}}\left(f\right)=10^{7.85}f^{-3.05}$$

Thus the FMCW beat signal from the mixer output with the inclusion of the signal phase noise is modeled as:

43 $$s_{3}\left(t\right)=a_{3}\cos\left[\phi \left(t\right)-\phi \left(t-T_{p}\right)+\delta \phi \left(t\right)-\delta \phi \left(t-T_{p}\right)\right]$$

Where *δϕ(t)* represents the phase noise phase at time *t*, *T*_*p*_ represents the difference in travel time on the parasitic signal path at the circulator and the travel time on the reference path from the signal generator to the mixer. In the event that the parasitic signal path and reference signal path are having the same travel time, their phase noise cancels out at that moment. The difference between the two process noises can be obtained using the transfer function model:

44 $$H\left(f,T_{p}\right)=1-e^{-j2\mathrm{\pi f}T_{p}}$$

Where one of the noise processes is a time-delayed function of the other. The phase noise process limiting the radar performance is modeled as:

45 $$\begin{array}{l} S_{\mathrm{phase}}\left(f,T_{p},L_{p},\right)=P_{t}10^{-\frac{L_{p}}{10}}{\left(\left|H\left(f,T_{p}\right)\right|\right)}^{2}S_{\mathrm{generate}}\left(f\right)\\ =P_{t}10^{-\frac{L_{p}}{10}}2\left[1-\cos\left(2\mathrm{\pi f}T_{p}\right)\right]S_{\mathrm{generate}}\left(f\right) \end{array}$$

The performance of the FMCW is limited by thermal noise having a power spectrum of

46 $$S_{\mathrm{thermal}}=10^{\mathrm{NF}/10}k_{B}T$$

Where *T* denotes the temperature of the radar in degrees Kelvin, *k*_*B*_ denotes Boltzmann’s constant (1.38 × 10^− 23^*JK*^− 1^), *NF* represents the noise factor in decibels. It is also an indication of the noise factor decibel level increase of the thermal noise above theoretical lower limit. The signal-to noise ratio of the FMCW radar echo is modeled as (Bradley 2009a):

47 $$\mathrm{SNR}=\frac{S_{\mathrm{echo}}\left(f,f_{\mathrm{beat}},B,T_{m}\right)T_{m}}{\left(\sum_{p=1}^{2}S_{\mathrm{phase}}\left(f,T_{p},L_{p}\right)+S_{\mathrm{thermal}}\right)}$$

The SNR is an indication of the power ratio of the sum of all limiting noise effects in the FMCW radar system having two parasitic phase noise paths. The detection of the radar echo is deemed reliable if the SNR is in the excess of 10 dB.

## FMCW radar performance prediction

FMCW radar performance may be viewed as a two- step process. The first step takes into account the estimation of the statistical properties of the radar signal and noise fields. The characteristic property in this analysis is the signal to noise ratio (SNR). The second step converts the SNR to probability of detection using an appropriate mapping that is suitable to the radar type and mode of operation. If the SNR can be accurately determined, then the probability of detection is a number that lies on the interval {0, 1}. In practice, the SNR is not known with total certainty.

Radar performance is predicted with aid of the standard radar equation. Expressing the SNR ratio as a normally distributed random variable χ, the probability density function is modelled as (Bradley 2009b):

48 $$h\left(x,\mu ,\sigma \right)=\frac{1}{\sqrt{2\pi \sigma }}\;\exp\left[-\frac{{\left(x-\mu \right)}^{2}}{2\sigma^{2}}\right]$$

The SNR as a log-normal distribution *y*, its probability density function is modelled as:

49 $$f\left(y,\mu ,\sigma \right)=\frac{1}{\sqrt{2\pi \sigma }}\;\frac{10}{\ln\left(10\right)}\;\frac{1}{y}\exp\left[-\frac{{\left(10\;\mathrm{lo}g_{10}\left(y\right)-\mu \right)}^{2}}{2\sigma^{2}}\right]$$

Where *x* and *y* have the relation,

50 $$Y=10^{\frac{X}{10}}\;,\;X=10\;\mathrm{lo}g_{10}\left(Y\right)$$

The probability density functions represent the same information about uncertainty in SNR on two intensity scale. The scales are 0 ≤ *y* ≤ *∞* and − *∞* < *x* < *∞*.

For optimisation of the radar having a signal with random phase and Rayleigh amplitude (Whalen 1971), the appropriate mapping SNR to probability of detection *P*_*D*_ is given as

51 $$P_{D}\left(y,P_{\mathrm{fa}}\right)={\left(P_{\mathrm{fa}}\right)}^{\frac{1}{1+y}}$$

With an inverse function of

52 $$v\left(P_{D},P_{\mathrm{fa}}\right)=\frac{\ln\left(P_{\mathrm{fa}}\right)}{P_{D}ln^{2}\left(P_{D}\right)}$$

The inverse function has simple properties that provide the platform of determining the probability density function *g*(*P*_*D*_*,P*_*fa*_*,μ,σ*) of the probability of detection *P*_*D*_ in closed form. The solution is given as

53 $$g\left(P_{D},P_{\mathrm{fa}},\mu ,\sigma \right)=-\frac{5\exp\left[-{\left(—\mu +\frac{10\ln\left[-1+\ln\left(P_{\mathrm{fa}}\right)/\ln\left(P_{D}\right)\right]}{\ln\left(10\right)}\;\right)}^{2}/2\sigma^{2}\right]\sqrt{\frac{2}{\pi }\;}\;\ln\left(P_{\mathrm{fa}}\right)}{\sigma \ln\left(10\right)ln^{2}\left(P_{D}\right)\left[-1+\ln\left(P_{\mathrm{fa}}\right)/\ln\left(P_{D}\right)\right]P_{D}}$$

For P_fa_ ≤ P_D_ ≤ 1. *σ* denotes the standard deviation of SNR (dB) and *μ* denotes the mean of SNR (dB).

As a result of the uncertainty in SNR, the probability of detection becomes a statistical distribution. The expected value and variability in the probability of detection are the useful characteristics of the statistical distribution. These characteristics are defined as:

54 $$\mu_{P_{D}}=\;E\left[P_{D}\right]=\overset{1}{\underset{P_{\mathrm{fa}}}{\int }}P_{D}\;g\left(P_{D},P_{\mathrm{fa}},\mu ,\sigma \right)\;dP_{D}$$

55 $$\begin{array}{ll} \;\sigma_{P_{D}}^{2} & =E\left[{\left(P_{D}-\mu_{P_{D}}\right)}^{2}\right]\\ =\overset{1}{\underset{P_{\mathrm{fa}}}{\int }}{\left(P_{D}-\bar{P_{D}}\right)}^{2}\;g\left(P_{D},P_{\mathrm{fa}},\mu ,\sigma \right)dP_{D} \end{array}$$

## Results

**Figure 2:**
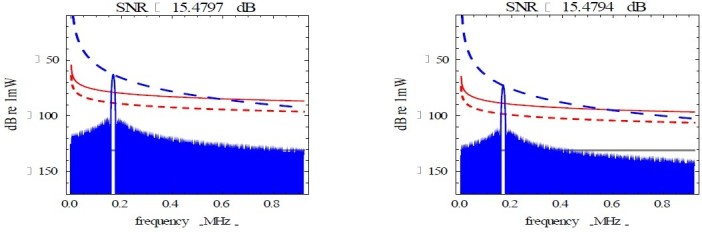
**Radar sweep analysis.** (**a**) Target Echo @ Reduced Sweep Width. (**b**) Target echo @ Increased Sweep Width.

**Figure 3:**
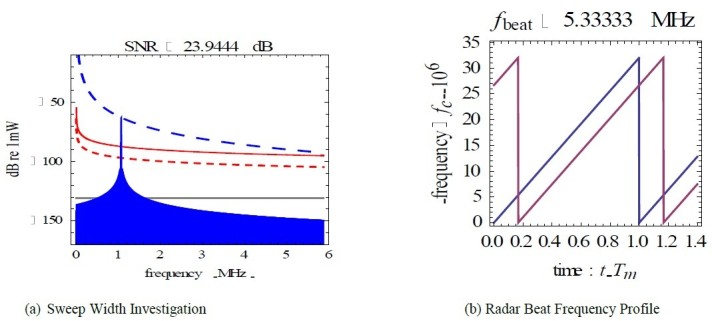
**Radar transmission power.** (**a**) Sweep Width Investigation. (**b**) Radar Beat Frequency Profile.

**Figure 4:**
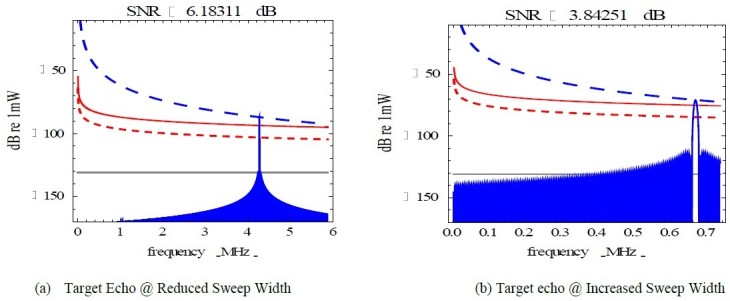
**Target echo analysis.**

The investigations on the FMCW radar revealed that the reduction of radar transmission power from 100 W to 10 W reduced the obstacle echo power linearly slightly below the noise factor level (NF) in accordance to the radar transmission power as shown in Figure 2-a and also reduced the frequency range of the obstacle echo frequency at the nominal sweep width of 5 MHz as illustrated in Figure 3. An increase in the sweep width of the radar resulted in an increase in the obstacle echo frequency. The radar beat frequency is attenuated at every second in a saw-tooth form shown in Figure 2-b. The further the obstacle, the less obstacle echo power was associated with the reduction in radar transmission power and radar sweep width as shown in Figure 4-a. Increasing the radar transmission power from 100 W to 1000 W and the radar sweep width increased the probability of detecting obstacles that are as far 5 km away as indicated in Figure 4-b.

**Figure 5:**
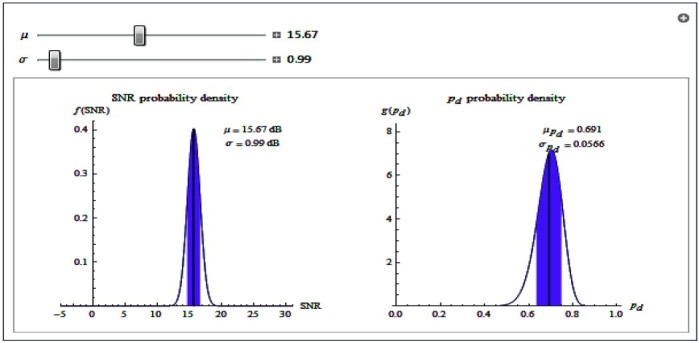
**Probability of detection having lower signal standard deviation.**

**Figure 6:**
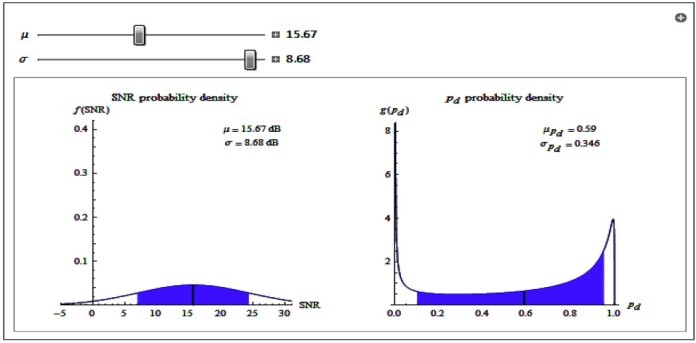
**Probability of detection having higher signal standard deviation.**

In the analysis of the probability of detecting targets as shown in Figures 5 and 6, the distribution of the probability of detection is peaked relatively small standard deviation when *σ* is small. This implies that the prediction of probability of detection is useful and meaningful in this case. With a larger *σ*, the distribution of the probability of detection is much spread out and has much higher standard deviation. The prediction in this case is not useful as it is an indication of greater uncertainty in the signal.

## Conclusion

The paper investigated the performance prediction model of an X-band 9410 MHz Furuno Radar emitting frequency modulation sweep at a 15 MHz short pulse and 5 MHz medium and long pulse repetition in obstacle detection and targeting tracking during maneuvering exercise of an USV. Obstacle range, radar cross section, the transmitting power of the radar and the gain of the radar antenna has noticeable effects on the performance of the radar in detecting and tracking obstacles. For effective performance of the radar to be achieved, it is proposed that the FMCW signal losses be reduced as much as possible and the time travel for the transmitted and received radar signal. The results showed that for a USV to be able to maneuver, detect and track targets effectively, the radar sweep width has to be increased to allow for narrower variability of radar signal and wider obstacle detection coverage and also increase in obstacle echo power and frequency.

## Authors’ original submitted files for images

Below are the links to the authors’ original submitted files for images. Authors’ original file for figure 1 Authors’ original file for figure 2 Authors’ original file for figure 3 Authors’ original file for figure 4 Authors’ original file for figure 5 Authors’ original file for figure 6
