# Supplementary figures and images for: Modelling the performance of USV manoeuvring and target tracking: an approach using frequency modulated continuous wave radar rotary system
Source: Springerplus. 2013 Apr 24;2(1):184. doi: 10.1186/2193-1801-2-184 (PMC3698431; doi:10.1186/2193-1801-2-184)

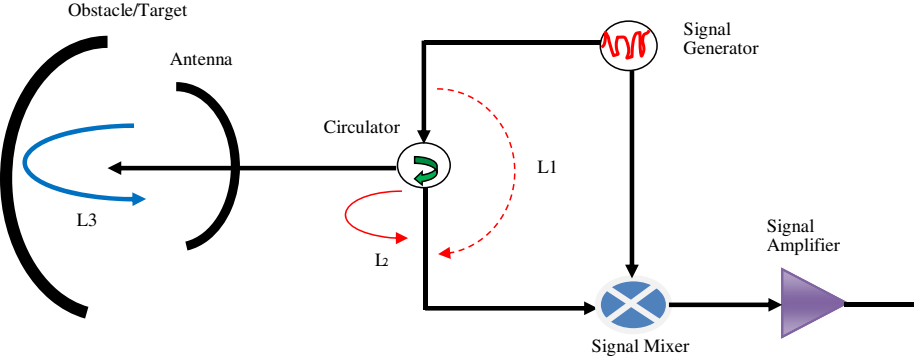

Supplement: Supplementary file 1 — Authors’ original file for figure 1 [file 40064_2012_343_MOESM1_ESM.pdf]

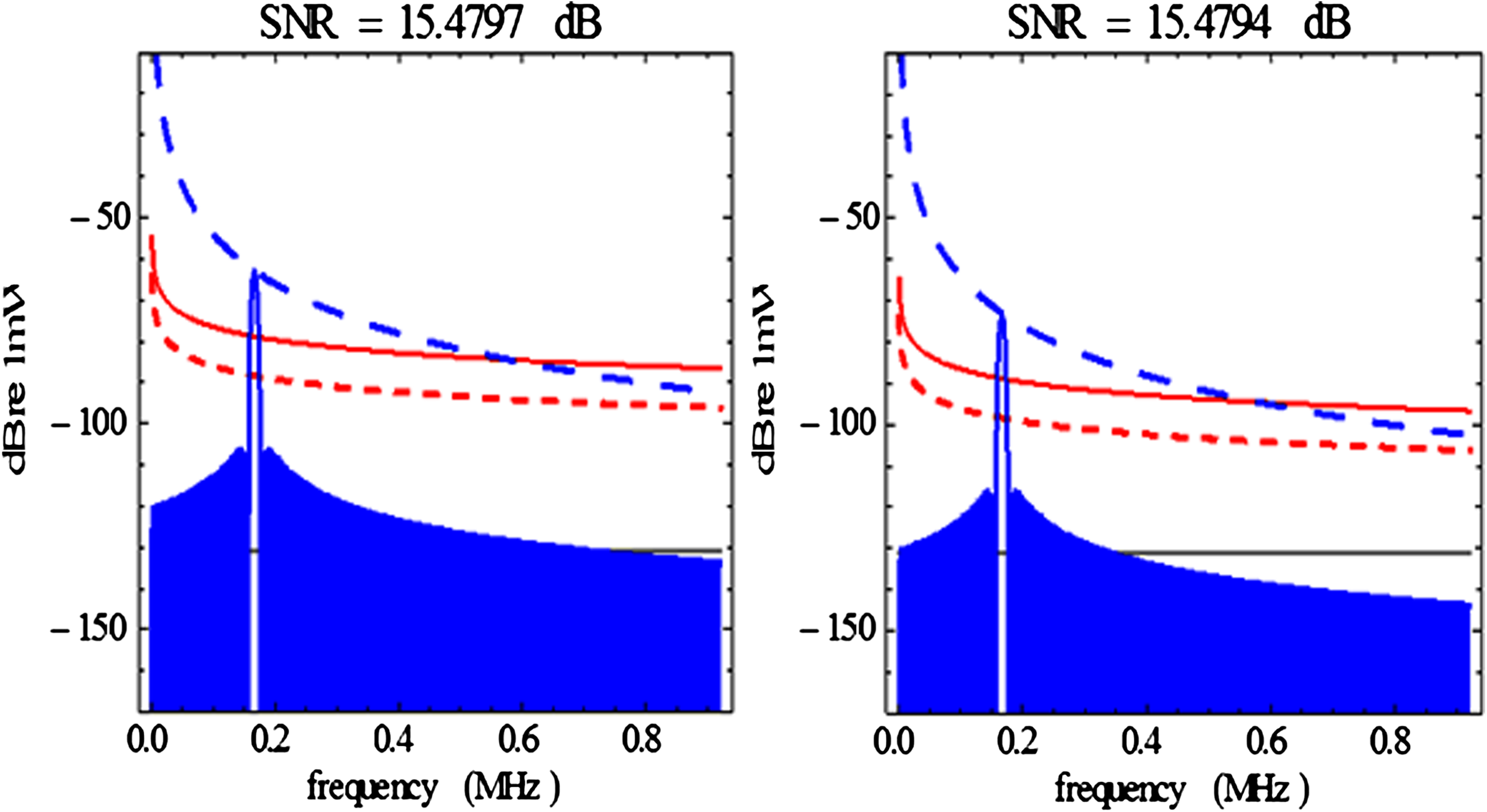

Supplement: Supplementary file 2 — Authors’ original file for figure 2 [file 40064_2012_343_MOESM2_ESM.tiff]

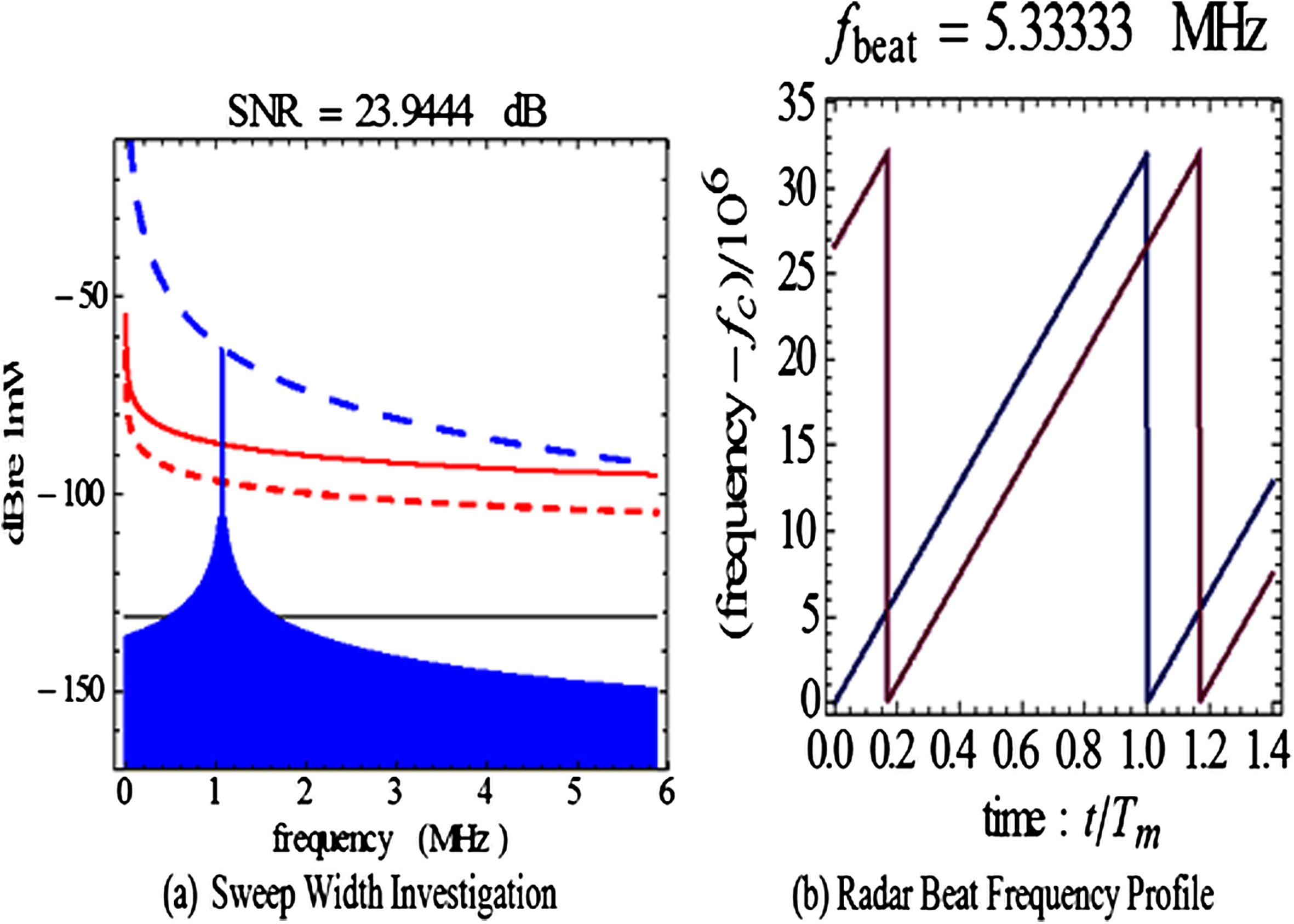

Supplement: Supplementary file 3 — Authors’ original file for figure 3 [file 40064_2012_343_MOESM3_ESM.tiff]

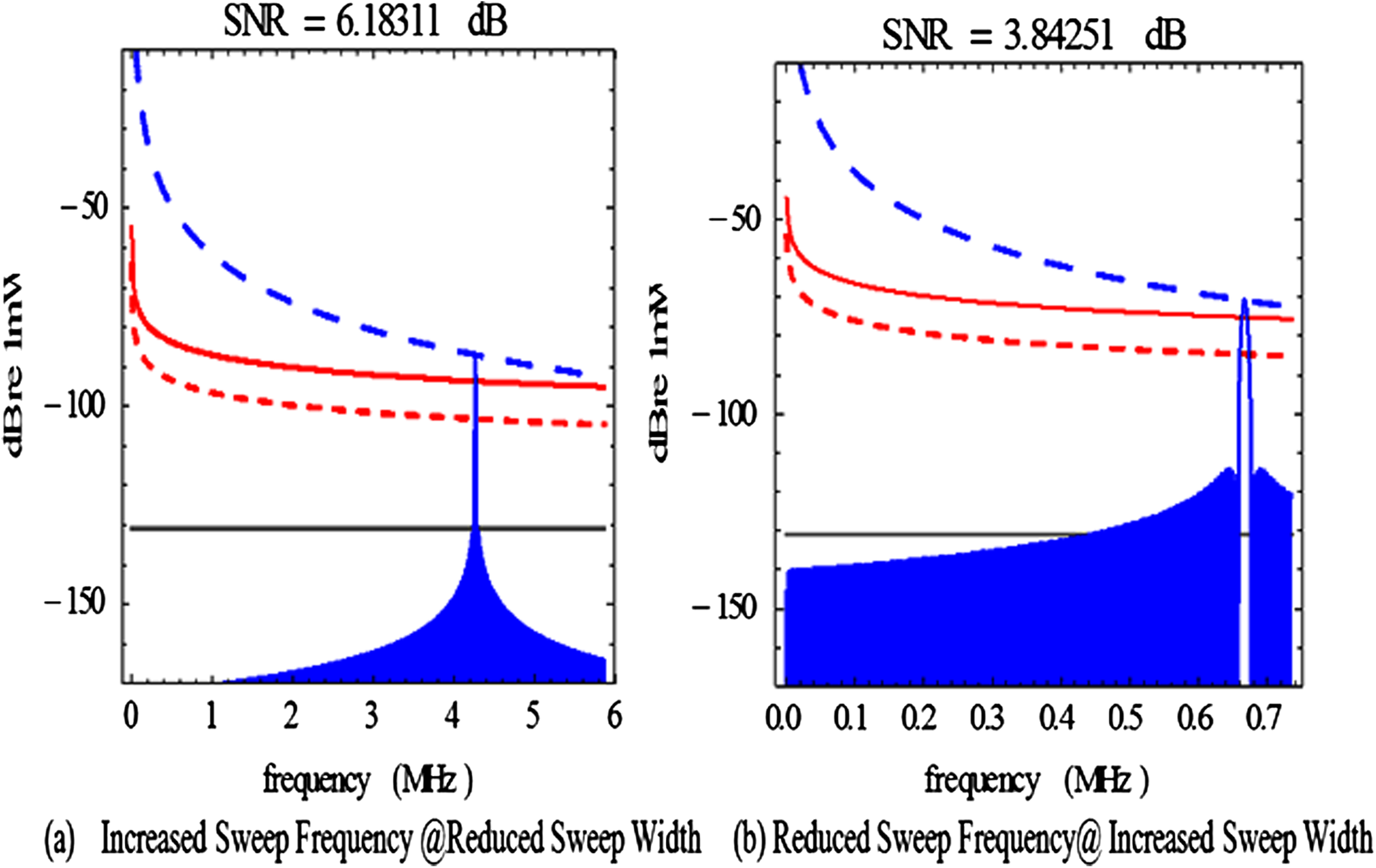

Supplement: Supplementary file 4 — Authors’ original file for figure 4 [file 40064_2012_343_MOESM4_ESM.tiff]

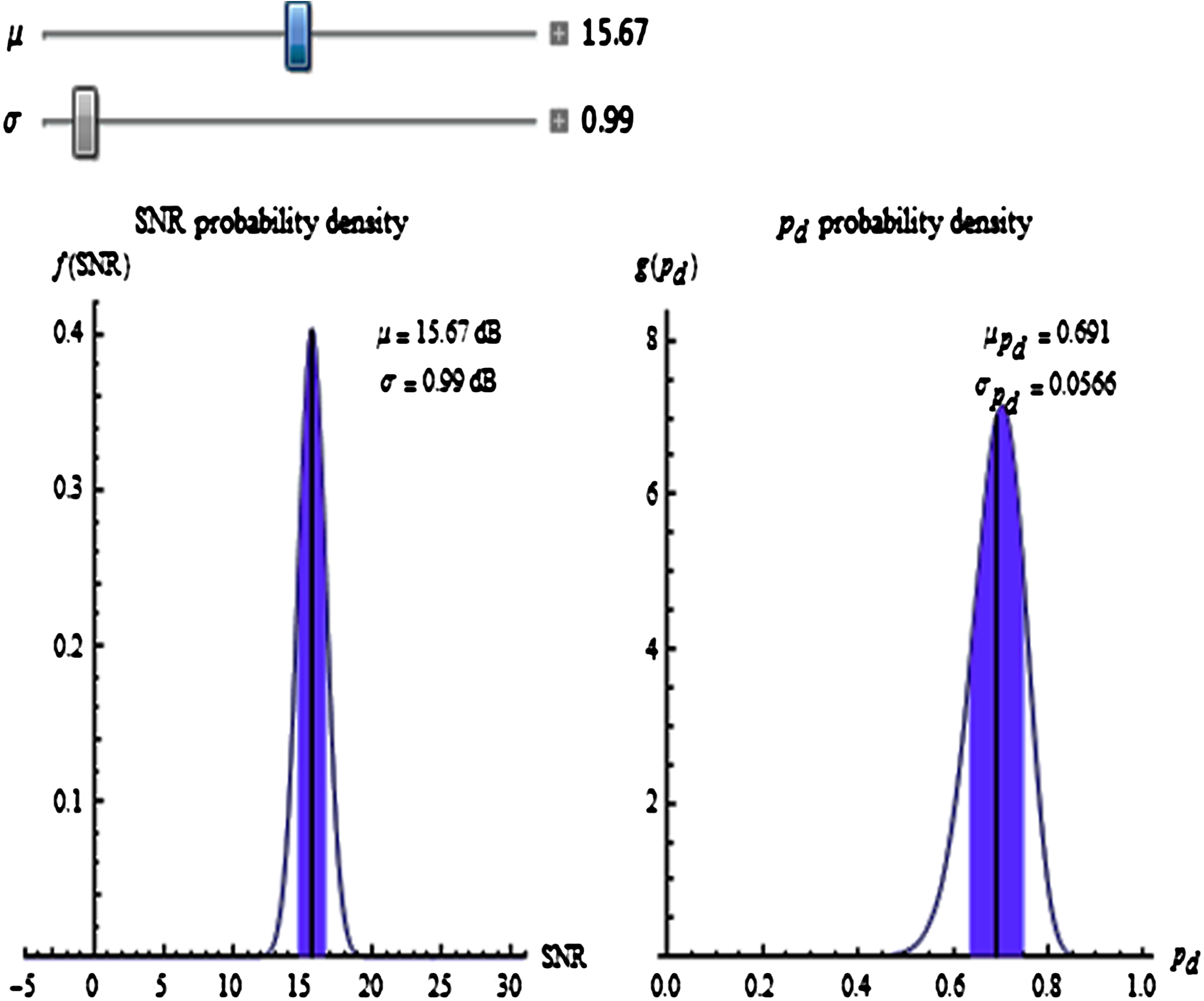

Supplement: Supplementary file 5 — Authors’ original file for figure 5 [file 40064_2012_343_MOESM5_ESM.tiff]

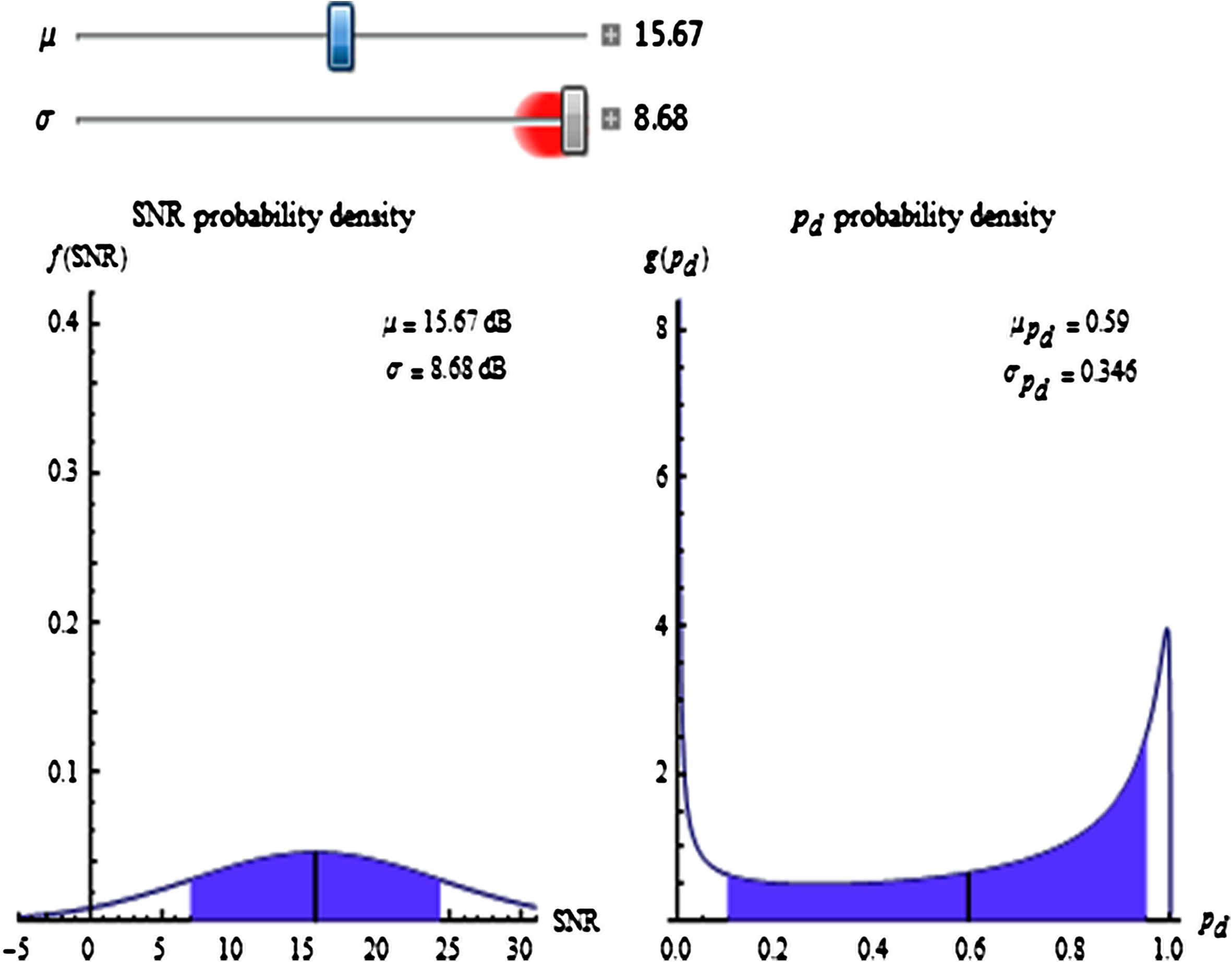

Supplement: Supplementary file 6 — Authors’ original file for figure 6 [file 40064_2012_343_MOESM6_ESM.tiff]
